# Voltage-gated ion channels mediate the electrotaxis of glioblastoma cells in a hybrid PMMA/PDMS microdevice

**DOI:** 10.1063/5.0004893

**Published:** 2020-07-01

**Authors:** Hsieh-Fu Tsai, Camilo IJspeert, Amy Q. Shen

**Affiliations:** Micro/Bio/Nanofluidics Unit, Okinawa Institute of Science and Technology Graduate University, Okinawa 904-0495, Japan

## Abstract

Transformed astrocytes in the most aggressive form cause glioblastoma, the most common cancer in the central nervous system with high mortality. The physiological electric field by neuronal local field potentials and tissue polarity may guide the infiltration of glioblastoma cells through the electrotaxis process. However, microenvironments with multiplex gradients are difficult to create. In this work, we have developed a hybrid microfluidic platform to study glioblastoma electrotaxis in controlled microenvironments with high throughput quantitative analysis by machine learning-powered single cell tracking software. By equalizing the hydrostatic pressure difference between inlets and outlets of the microchannel, uniform single cells can be seeded reliably inside the microdevice. The electrotaxis of two glioblastoma models, T98G and U-251MG, requires an optimal laminin-containing extracellular matrix and exhibits opposite directional and electro-alignment tendencies. Calcium signaling is a key contributor in glioblastoma pathophysiology but its role in glioblastoma electrotaxis is still an open question. Anodal T98G electrotaxis and cathodal U-251MG electrotaxis require the presence of extracellular calcium cations. U-251MG electrotaxis is dependent on the P/Q-type voltage-gated calcium channel (VGCC) and T98G is dependent on the R-type VGCC. U-251MG electrotaxis and T98G electrotaxis are also mediated by A-type (rapidly inactivating) voltage-gated potassium channels and acid-sensing sodium channels. The involvement of multiple ion channels suggests that the glioblastoma electrotaxis is complex and patient-specific ion channel expression can be critical to develop personalized therapeutics to fight against cancer metastasis. The hybrid microfluidic design and machine learning-powered single cell analysis provide a simple and flexible platform for quantitative investigation of complicated biological systems.

## I. INTRODUCTION

Glioma is one of the most common types of brain cancer and the aggressive form of it, glioblastoma, contributes to poor prognosis, high mortality, and high probability of recurrence,^1,2^ due to the infiltration nature of the disease. The highly infiltrative ability of glioblastoma originates from the invasive/migratory ability of glioma stem cells or brain tumor initiating cells.^3,4^ Not only glioma cells are important, but also the microenvironment in the brain helps shaping the heterogeneity of the glioma.^5^ The glioma cells interact with the extracellular matrix (ECM), glial cells, and immune cells in the brain and mediate the formation of peri-vascular, peri-necrotic, and invasive tumor microenvironments.^6–9^ Understanding the molecular mechanisms of the invasiveness in glioma cells with respect to the tumor microenvironment is vital for developing new therapeutic options and improving the patient outcome.^10,11^

In the brain, glial cells are immersed in an electric field created by tissue polarity from brain macrostructures as well as the local field potentials, which are established from the action potentials fired by the neurons.^12^ A weak endogenous electric current has been shown to serve as a guidance cue for neuroblast migration from the subventricular zone in mice,^13^ a region speculated as the origin of glioma tumorigenesis.^14^ Thus, the physiological electric fields in the brain may play an important role in mediating the glioma tumorigenesis and invasion.^15–19^ Cells sense the electric field by bioelectrical activation of voltage-sensitive proteins, mechanosensing due to electrokinetic phenomena, or activated chemical signaling due to electrokinetically polarized membrane receptors (Fig. S1). The voltage gradient creates a large voltage drop at the cellular membrane, which can directly activate voltage sensitive proteins such as voltage-gated ion channels that are most commonly expressed on excitable membranes at neuronal synapses and neuro-muscular junctions.^20^ Among the voltage-gated ion channels in the brain, calcium channels are especially important as calcium influx plays a pivotal role in cellular signaling.^21,22^ Calcium signaling is also important in glioma cell proliferation, resistance to therapy, and metastasis.^23–27^ Whether or not the calcium signaling in glioma is mediated by electric field is still an open question.

**FIG. 1.**
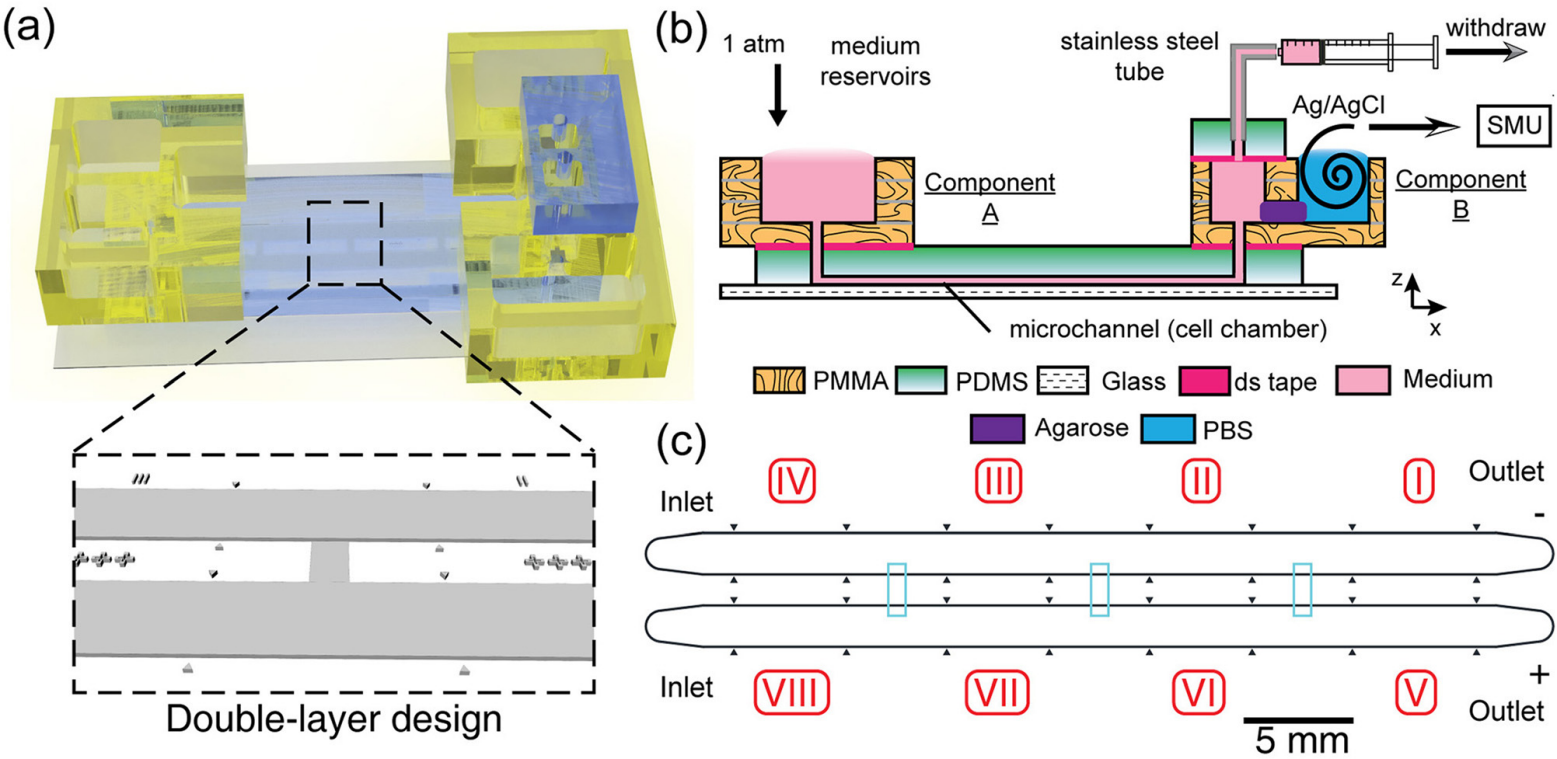
The design of a hybrid multiple electric field chip (HMEFC). (a) the 3D rendered model of the final HMEFC. Double-layer microchannel design in the PDMS is shown; (b) the schematic diagram of using the HMEFC for electrotaxis experiments. Complex 3D microfluidic structures and world-to-chip interface are established in PMMA components A and B. Simplified chemical infusion can be performed using the medium reservoirs in the Component A. Electrical stimulation with salt bridge separation is integrated in the Component B; (c) the channel design. The 10 *μ*m-tall first layer structures are shown in cyan. Multiple electric fields are created in Secs. I–IV and V–VIII with a strength ratio of 5.25:2.5:1:0. Two chemical conditions can be introduced to Secs. I–IV and V–VIII from the two inlets with minimal cross-contamination.

Conventional *in vitro* electrical stimulation systems for studying cell responses in electrical microenvironments are bulky and the experimental throughput is limited.^28,29^ To overcome these limitations, a robust high-throughput *in vitro* platform that creates stable electrical stimulation of cells and interfaces with automated microscopy is a prerequisite for rapid screening of targets and identifying molecular mechanisms. To this end, we have developed a hybrid poly(methylmethacrylate)/poly(dimethylsiloxane) (PMMA/PDMS) microfluidic platform to reliably study glioblastoma single cell migration under multiplex dcEF stimulations that multiple antagonists can be tested simultaneously to identify molecular mechanisms. Quantitative single cell migration analysis is carried out by extracting cell migration metrics such as the directedness, orientation, and speed using robust machine learning-powered cell segmentation/tracking/analysis software with stain-free phase contrast microscopy.^30^ Using the hybrid microfluidic platform, the role of voltage-gated calcium channels in calcium signaling pathways of glioblastoma electrotaxis is investigated.

## II. RESULTS

### A. Multiplex electrotaxis experiments using a hybrid multiple electric field chip (HMEFC)

To investigate cell electrotaxis and elucidate molecular mechanism in different phenotypes, a reliable *in vitro* multiplex platform is required. The hybrid multiple electric field chip (HMEFC) is designed by the hybrid PMMA/PDMS approach^17^ [Fig. 1(a)]. Using PMMA-based microfluidics, a versatile world-to-chip interface and 3D designs can be easily integrated. Medium reservoirs (Component A) and interface for electrical current introduction and medium withdraw (Component B) can be easily fabricated using laser cutting and thermal bonding of PMMA substrates [Fig. 1(b)]. In PDMS-based microfluidics, by using double-layer microchannel design, the fluid and chemical transport in a controlled microenvironment can be realized for studying cell electrotaxis. An experiment with two chemical stimulations with four electric field strengths of a ratio of 5.25:2.5:1:0 can be performed on a single HMEFC, increasing the experimental throughput^15,31,32^ [Fig. 1(c)], see more discussion in the supplementary material.

### B. Uniform single cell seeding by submerged manipulation in a hybrid multiple electric field chip (HMEFC)

To analyze single cell migration in microchannels, cells must be seeded sparsely and allowed to adhere and culture reliably.^33,34^ However, it is known that uneven distribution of cells due to fluid flow, convection in suspension, and vessel movement after seeding can cause aggregation and differentiation of cells.^35^ In particular, seeding cells with minimum pressure difference in a microfluidic device for reproducible single cell studies is challenging. In this work, we designed a removable top reservoir on top of a microfluidic chip to balance the hydrostatic pressure during cell seeding so human errors can be reduced.

**FIG. 2.**
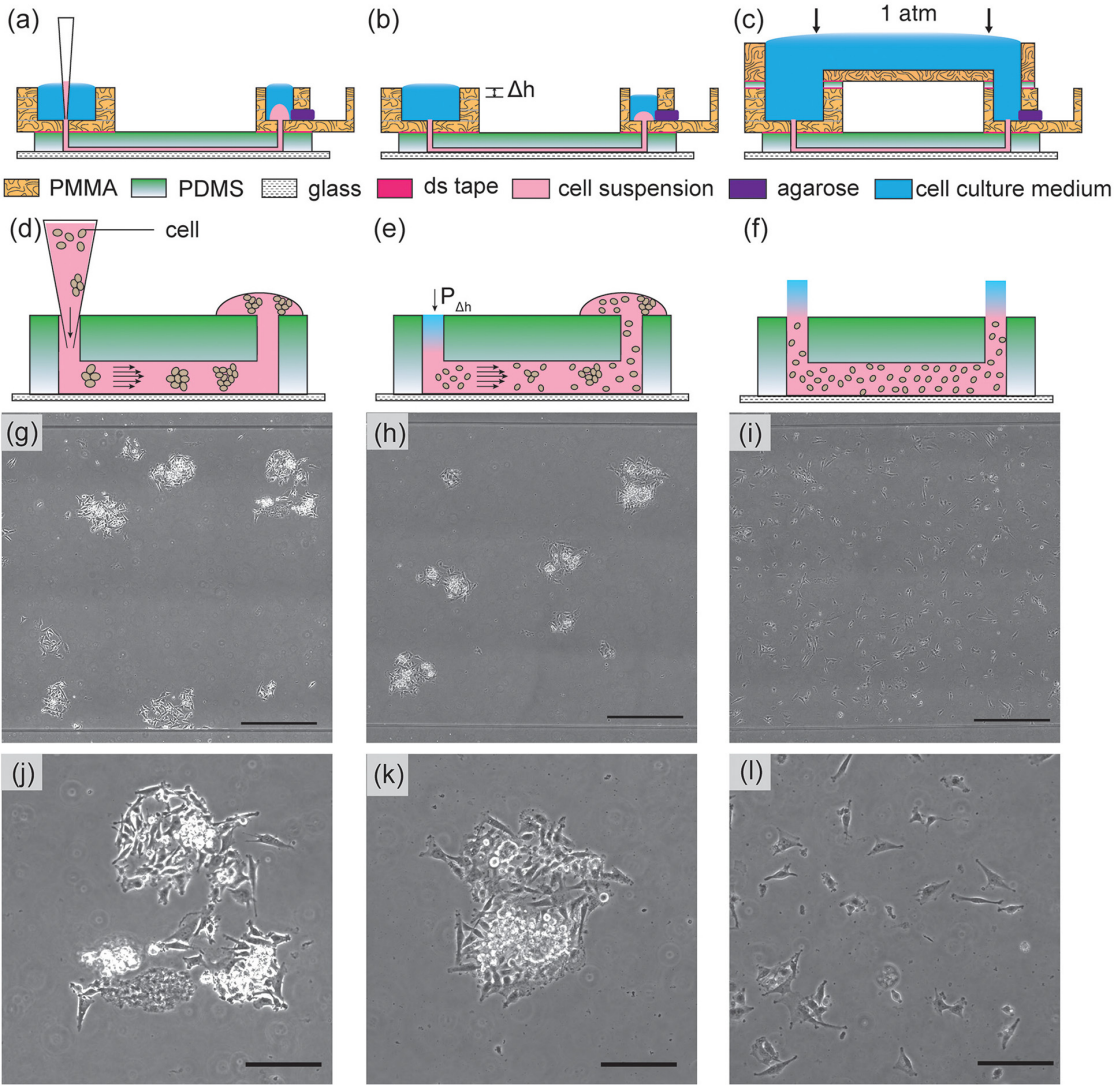
The results of U-251MG cell seeding inside microchannels by various methods. [Left column: (a), (d), (g), and (j)]. In the tip loading method, cells are introduced by using gravitational flow with micropipette tips. The cells can flocculate inside the tips and in microchannels as illustrated in (d). The microscopy image of seeded cells is shown in (g) and magnified in (j); [Middle column: (b), (e), (h), and (k)] in the tip injection method, cells are injected into the channels and tips are removed. The small hydrostatic pressure differences between the inlet/outlet (shown as Δh) will contribute to hydrodynamic flow and disturb the cell distribution, causing non-uniform cell distribution and aggregates as shown in (e). The microscopy image of seeded cells is shown in (h) and magnified in (k); [Right column: (c), (f), (i), and (l)] in our pressure-balanced submerged cell seeding method, the hydrostatic pressure difference is eliminated. The injected cells remain uniform throughout the channel as shown in (f). The microscopy image of seeded cells is shown in (i) and magnified in (l). The uniform and sparse cell seeding method is suitable for different applications such as single cell tracking, ensembled cell studies, and cell assembly. The scale bars in [(g), (h), and (i)] represent 500 *μ*m. The scale bars in [(j), (k), and (l)] represent 200 *μ*m.

Different cell loading methods affecting cell distribution in single cell migration experiments are investigated, such as the tip loading method, tip injection method, and a pressure-balanced submerged cell seeding, as shown in Fig. 2.

In the tip loading method [Fig. 2(a)], the cells flocculate in the small pipet tip and cannot be dispersed uniformly in the microchannel [Figs. 2(d), 2(g), and 2(j)]. In the tip injection method [Fig. 2(b)], the cells are originally injected in the channels with uniform cell distribution. However, without balancing the microchannel inlet/outlet pressure, the minute hydrostatic pressure difference between inlets and outlets generates a small pressure-driven flow that displaces cells, which lead to cell aggregates [Figs. 2(e), 2(h), and 2(k)]. Furthermore, in the tip injection method, due to the small dimension of the punched holes at inlet/outlet interfaces, bubbles are easily trapped and may be introduced into microchannels, disrupting fluid advection and chemical transport.

By submerging inlets and outlets underwater using a reversibly bonded top reservoir and balancing the pressure between inlets and outlets [Figs. 2(c)], air bubbles can be avoided and pressure-driven flow is prevented from affecting cell distribution. Moreover, using this cell seeding method, only a minute amount of cells is needed (the volume of the microchannel). Uniformly distributed single cell seeding across the entire microchannel is obtained for single cell migration experiments [Figs. 2(f), 2(i), and 2(l)]. The top reservoir in our submerged cell seeding setup can be easily removed after cells are seeded and can be adapted to a wide range of microfluidic chips.

### C. Glioblastoma electrotaxis requires an optimal laminin-containing ECM

An effective ECM coating on the substrate is essential for cell adhesion and formation of focal adhesions for cell migration.^36,37^ Glioblastoma can be molecularly classified into proneural, neural, classical, and mesenchymal types according to The Cancer Genome Atlas (TCGA).^38^ We use two glioblastoma cell models, T98G, and U-251MG, which are both of caucasian male origin and classified as mesenchymal type with p53 mutant genotype.^39,40^ The adhesion and electrotaxis of T98G and U-251MG glioblastoma cell lines on various ECMs are tested in a double-layer hybrid multiple electric field chip (HMEFC) based on the hybrid PMMA/PDMS design approach^17^ (Table S1). The migration directedness, speed, and morphology of glioblastoma cells (see details in Sec. IV E) are quantitatively analyzed by custom-made machine learning-based single cell segmentation and tracking software from stain-free phase contrast microscopy.^30^ The cell morphologies of the two cell lines on various ECMs are shown in Fig. S2.

While standard poly(D-lysine) (PDL) and various combinations of poly(L-ornithine) (PLO) and laminin have been used for glioblastoma electrotaxis,^16,41^ the adhesion and electrotaxis of T98G and U-251MG are not always consistent and reproducible as shown in Figs. S2 and S3. T98G and U-251MG electrotactic responses are also not stably reproduced on collagen I, collagen IV, vitronectin, and fibronectin coatings.

**FIG. 3.**
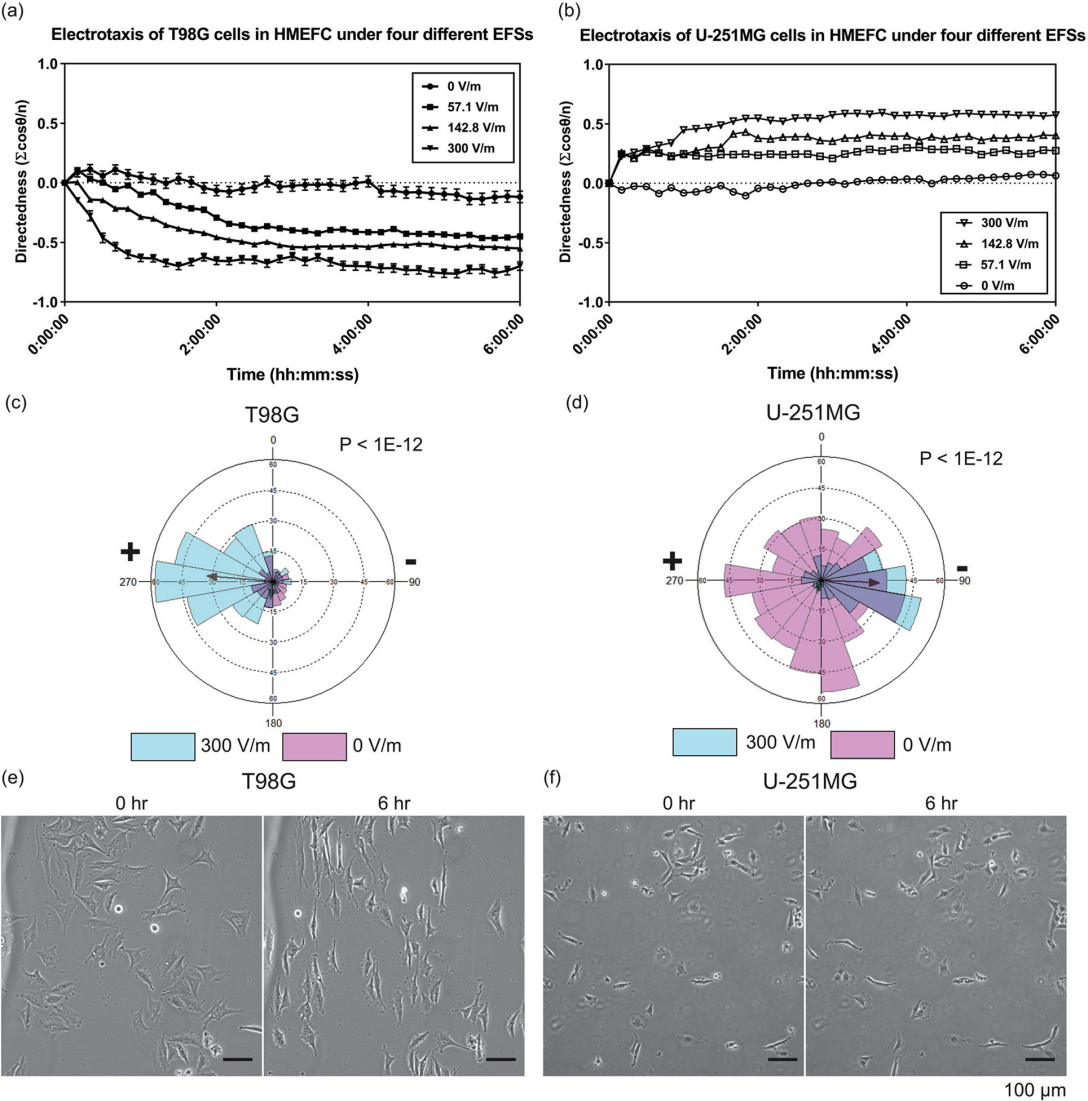
The electrotaxis and electro-alignment of T98G and U-251MG glioblastoma cells in HMEFC. (a) The electrotaxis directedness of T98G cells in four different electric field strengths (EFSs); (b) the electrotaxis directedness of U-251MG cells in four different EFSs; [(c) and (d)] The rose plots showing the frequency of T98G (c) and U-251MG (d) electrotaxis with and without electric field. The r-vectors displaying the direction tendencies of each group are indicated by arrows; P values in Mardia–Watson–Wheeler tests are shown; (e) phase contrast images of T98G and (f) U-251MG cells before and after 6 h under 300 V m^−1^ stimulation; the electric field is applied from left to right. Only T98G cells demonstrate prominent perpendicular alignment under the electrical stimulation.

Both T98G and U-251MG cells adhere well and demonstrate lamellipodia structures on substrates containing laminins, such as pure laminin coating, or Geltrex.^TM^ Geltrex^TM^ is a growth factor-reduced complex basement membrane extract purified from murine Engelbreth–Holm–Swarm tumors containing laminin, collagen IV, entactin, and heparin sulfate proteoglycan.^42^ Cells interact with laminins through various integrins including *α*1*β*1, *α*2*β*1, *α*3*β*1, *α*6*β*1, and *α*7*β*1.^43,44^ The integrins are believed to participate in the initiation of electrotaxis through mechanosensitive pathways.^45–47^

The typical *in vitro* electric fields are in the range of 100–300 V m^−1^ among glioblastoma, neural stem cells, and other solid tumor cell electrotaxis studies.^16,48–51^ Similar electric field strength has also been used for therapeutic tumor treating pulsed electric field.^18^ Based on this information, using our hybrid multiple electric field chip (HMEFC), four electric field strengths (0, 57.1, 142.8, and 300 V m^−1^) are created for simultaneous screening of cell behavior in four different electric field strength (EFSs). Note that our selected EFS strength is greater than the physiological electric field measured along the rostral migratory stream in the mice brain (around 3 V m^−1^), though the electric field in human brains may be larger.^13^

Discussed in detail in Sec. IV D, under electrical stimulation, the electrotaxis of both T98G and U-251MG is more prominent and reproducible on Geltrex^TM^ coatings; hence, all the studies in the following sections are based on Geltrex^TM^ coatings. The detailed data of T98G and U-251MG electrotaxis on various ECMs are shown in Table S2 of the supplementary information.

An interesting observation is found in U-251MG cells on iMatrix-511-coated substrates. U-251MG cells demonstrate large lamellipodia associated with high migratory speed (15.45 *μ*m h^−1^ under 300 V m^−1^, P < 0.0001) but with diminished directedness (0.01, P < 0.0001). Note that iMatrix-511 is a recombinant truncated laminin with *α*5*β*1*γ*1 subunits and interacts with cells through the *α*6*β*1 integrin.^52,53^ This suggests that the specific molecular configuration of laminins in ECM may be vital for electrotaxis.

While it is understood that ECMs in the tumor microenvironment are important,^54^ glioblastoma cells demonstrate preference for adherence and electrotaxis on laminin-coated surfaces. Within the brain microenvironment, laminin expressions are restricted to the basement membrane of neural vasculature^55^ and a perivascular tumor microenvironment is especially vital for glioblastoma metastasis.^10,56^ Therefore, the correlation among laminin, glioblastoma electrotaxis, and the perivascular invasion process may be important in glioblastoma cancer biology that requires further elucidation.

### D. Electrotaxis behavior may reflect the heterogeneity of glioblastoma

To further analyze how T98G and U-251MG cells migrate under dcEF stimulation, the glioblastoma electrotaxis in serum-containing (FBS) and serum-free media was examined.

#### 1. T98G and U-251MG cells migrate toward opposite directions under dcEF stimulation

Figures 3(a) and 3(b) display the directedness of T98G and U-251MG electrotaxis. For the range of dcEF stimulations applied (57.1–300 V m^−1^), T98G cells migrate toward the anode (positive electrode) while the U-251MG cells migrate toward the cathode (negative electrode), and the directedness increases with increasing dcEF. Furthermore, the directedness in the electrotaxis of T98G cells is not affected by the presence of fetal bovine serum (FBS) (P=0.45) but the speed is significantly decreased (P<0.0001) (Fig. S8). However, the directedness of U-251MG cell electrotaxis is highly dependent on the presence of FBS (P < 0.0001) and the speed of U-251MG electrotaxis increases with FBS (P < 0.0001) (Fig. S8). The fetal bovine serum is rich in growth factors, proteins, and ions, which can enhance the chemical signaling in electrotaxis (Fig. S1). The electrotaxis and random migration of T98G and U-251MG with or without electrical stimulation are shown in Videos S1–S4.

Both T98G cells and U-251MG cells are categorized as mesenchymal type glioblastoma;^39,40^ however, their electrotactic responses are completely different. Similar results are reported in the electrotaxis of other glioblastoma cells and spheroids^15,16,19,41^ as well as in lung adenocarcinoma cells,^31^ showing that although cell lines can have similar molecular and surface marker makeups, their electrotaxis responses can be completely different. To better illustrate the directionality of T98G and U-251MG electrotaxis, directional migration frequencies of T98G [Fig. 3(c)] and U-251MG [Fig. 3(d)] electrotaxis are shown in rose plots.

Furthermore, the average size of T98G cells is larger than that of U-251MG cells and the size of T98G cells also increases in the first 3 h of electrotaxis (Fig. S4), reflecting rapid cell morphology changes during the electrotaxis. It is also observed that an enlarged multinuclei subpopulation exists in T98G cells and these multinuclei cells exhibit weak senescence-associated *β*-galactosidase activity (Fig. S5). The multinuclei cells are also electrotactic (Video S1) and associated with drug resistance.^57,58^ In contrast, U-251MG cells are less electrotactic and not all U-251MG cells respond to the electric field (Video S3). The very different electrotaxis responses of the two cell lines may reflect the fundamental cell heterogeneity of glioblastoma which has been speculated to contribute to the recurrence and therapeutic resistance after anti-tumor therapy.^59–64^

**FIG. 4.**
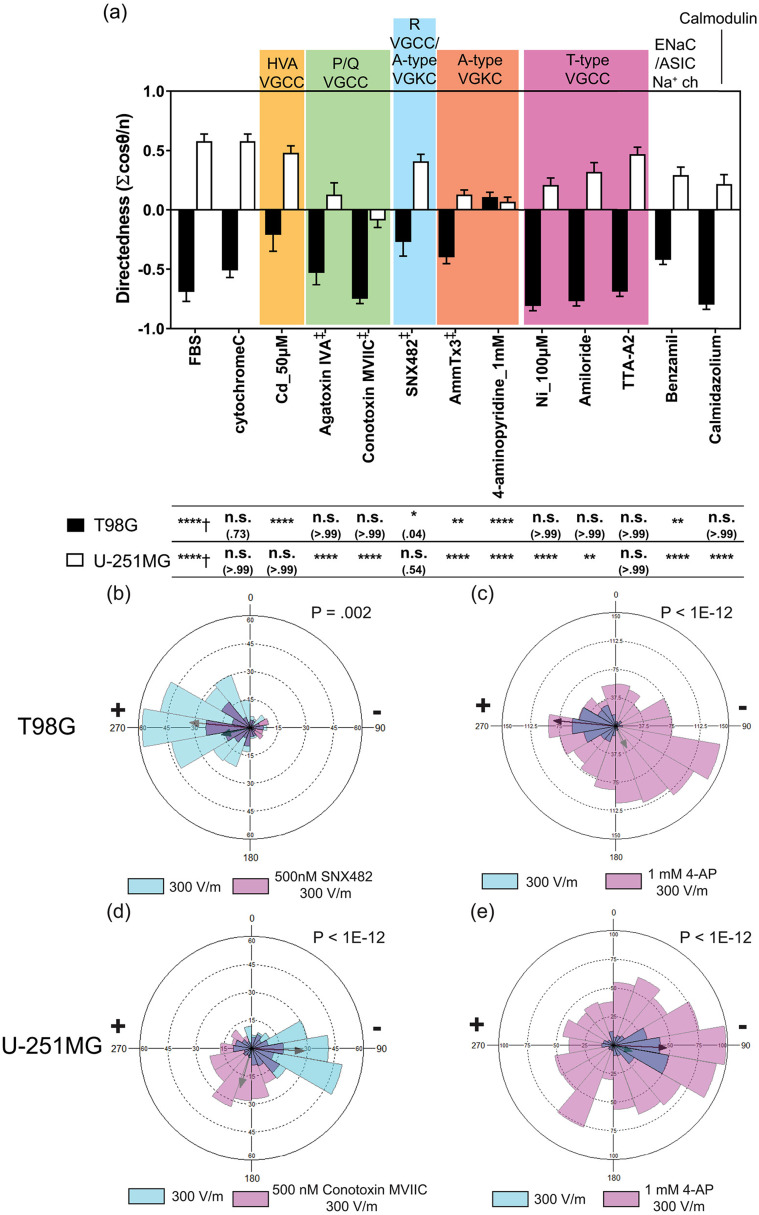
Inhibitory effects of selected ion channels on glioblastoma cell electrotaxis. (a) The electrotactic directedness of T98G and U-251MG glioblastoma cells under 300 V m^−1^ dcEF after 6 h with pharmacological inhibition on various ion channels. † indicates the electrotaxis tested against those without EF stimulation; ‡ indicates the electrotaxis group tested against those with cytochrome C which prevents adsorption of short peptides to experimental apparatus; All other groups are statistically compared to their respective controls in cell culture media with 10% FBS; n.s. indicates not significant; * indicates P < 0.05; ** indicates P < 0.01; *** indicates P < 0.001; **** indicates P < 0.0001; the numbers in parentheses indicate actual P-values; [(b) and (c)] Rose plots showing the frequency of T98G electrotaxis with and without the presence of SNX482 (R-type VGCC inhibitor) and 4-AP (A-type VGKC inhibitor); the r-vectors displaying the direction tendencies of each group are indicated by the arrows; P values in Mardia–Watson–Wheeler tests are shown; (d) and (e) rose plots showing the frequency of U-251MG electrotaxis with and without the presence of *ω*-conotoxin-MVIIC (P/Q-type VGCC inhibitor) and 4-AP; The r-vectors displaying the direction tendencies of each group are indicated by the arrows; P values in Mardia–Watson–Wheeler tests are indicated. Note that the scale in [(b) and (d)] is different from that in [(c) and (e)] due to different numbers of cells analyzed. The histograms represent the frequencies of cells moved in each direction.

**FIG. 5.**
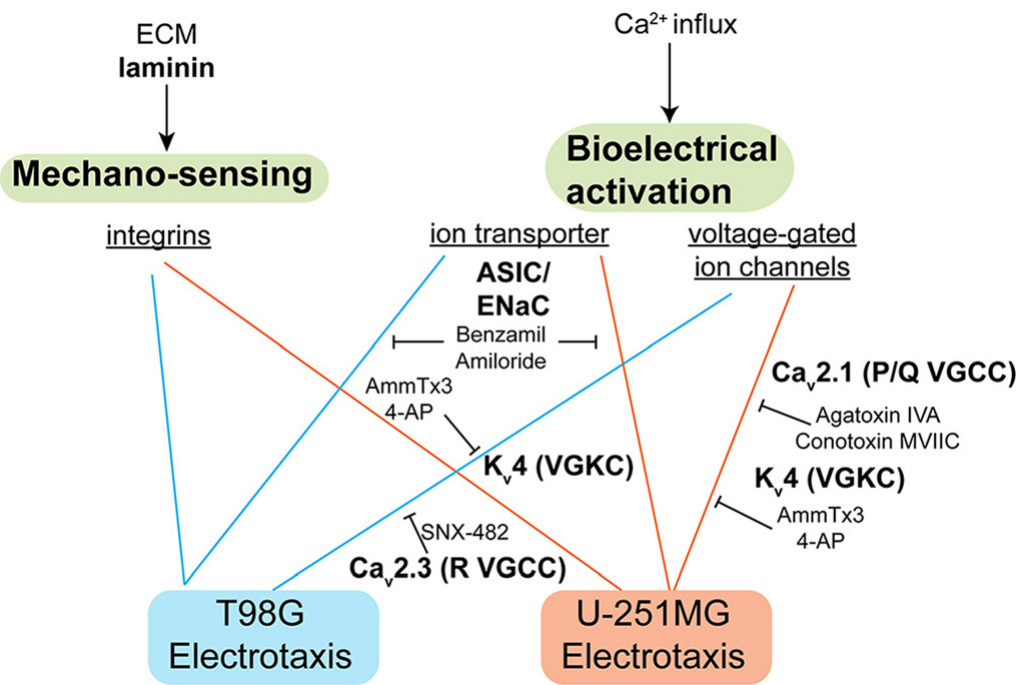
The signaling mechanism identified in this study. Laminin-based ECMs is necessary for glioblastoma electrotaxis, suggesting that integrins may play a role. The voltage-gated ion channels and ion transporters also mediate glioblastoma electrotaxis that requires extracellular calcium.

Further elucidation of the correlation among electrotactic responses, metastatic properties of glioblastoma, and *in situ* electric field around the lesion may be beneficial to evaluate electrotaxis response as a predictive tool for glioblastoma metastasis.

#### 2. Only T98G cells demonstrate prominent electro-alignment behavior under electrical stimulation

Aside from directional migration in the dcEF, cells may also demonstrate long axial alignment in perpendicular to the dcEF vector. While this phenomenon is commonly observed among many cell types,^31,65–68^ the molecular mechanism and the biological role are not clear. Electro-alignment may participate in the cytoskeletal restructuring in tissue morphogenesis,^69,70^ but biophysical studies show that *in vitro* microtubules align in parallel to electric field vectors rather than perpendicular.^71,72^

Figure S6 shows orientation of the cells with respect to time in electrically stimulated T98G and U-251MG cells over 6 h. The orientation index is defined as the average cosine of two times the angle between the long axis of a cell and the electric field vector (See more details in Fig. S13 and Table S5). For a group of perpendicularly oriented cells, the average orientation index is −1 and for a group of parallely orientated cells, the average orientation index is 1. For a group of randomly arranged cells, the average orientation index should be 0. Only T98G cells show prominent perpendicular alignment after electrical stimulation (${\text{Index}}_{\text{orientation}}$ at 0 h vs 6 h is −0.11 vs −0.83, P < 0.0001). FBS deprivation slightly decreases the alignment tendency and delays the onset but does not abolish it (${\text{Index}}_{\text{orientation}}$ of serum free vs 10% FBS at 6 h is −0.48 vs −0.83, P < 0.0001). However, U-251MG does not show any perpendicular alignment. These results further illustrate the heterogeneity of glioblastoma cells. The cell electroalignment phenotypes of T98G and U-251MG after dcEF stimulation are shown in Figs. 3(e) and 3(f).

### E. Glioblastoma electrotaxis requires extracellular calcium

Calcium ion flux is known to be involved in the electrotaxis signaling of multiple cell types including mouse fibroblasts, human prostate cancer cells, and neural crest cells.^73–77^ Deregulation of calcium influx in the cells reduces actin polymerization and affects cell motility speed but its effect on electrotactic directedness varies depending on cell types.^73,77^ The hypothesis that glioblastoma electrotaxis is dependent of extracellular calcium cations is tested in this work (Fig. S8).

First, a calcium-free, serum-free cell culture medium (CaFree SF) is used to test if glioblastoma electrotaxis requires extracellular calcium. T98G cells lose viability both with and without dcEF stimulation [Figs. S8(a) and S11]. The lack of calcium ions in cell culture media may impact calcium homeostasis significantly but the loss of viability is rescued by the addition of 10% FBS, which contains calcium and growth factors. The electrotaxis of U-251MG in calcium-free, serum-free media is not affected compared to those in serum-free media (P > 0.99). Interestingly, the electrotactic speed of U-251MG in calcium-free serum-free media is greater (P < 0.0001).

Second, to validate that calcium cations are important, cation chelators ethylenediaminetetraacetic acid (EDTA) and ethylene glycol-bis(2-aminoethylether)-N,N,N′,N′-tetraacetic acid (EGTA) are used to chelate the free calcium in the cell culture media with 10% FBS. Treatment of 2 mM EDTA significantly suppresses the directedness of only U-251MG cells [P < 0.0001, Fig. S8(c)]. To further confirm that the electrotaxis inhibition is due to extracellular calcium cations, EGTA, a divalent cation chelator with increased affinity toward Ca^2+^, is used. At 1 mM EGTA, the electrotactic directednesses of neither cell lines is affected but their electrotactic speeds become more dispersed (P < 0.0001). Under 2 mM EGTA treatments, the electrotactic directedness of U-251MG cells is reduced (P < 0.0001). When 5 mM EGTA is used, the electrotaxis of both cell lines is further repressed in both directedness and speed and the cells detach from the substrate. These results suggest that glioblastoma electrotaxis requires extracellular calcium cations and calcium influx may be important for electrotaxis, particularly that of U-251MG cells.

### F. Glioblastoma electrotaxis is mediated by voltage-gated ion channels

Ions channels expressed on glioblastoma cells including various potassium, calcium, sodium, and chloride ion channels are believed to facilitate pathogenesis of glioblastoma.^27,78–80^ Although the expressions of numerous ion channels vary among clinical glioma samples,^25^ ion channel expression profiles have been suggested to predict survival in glioma patients.^81,82^ Glioblastoma cells are immersed in the local field potentials within the brain, and extracellular calcium is required for glioblastoma electrotaxis which can flow into the cells through voltage-gated calcium channels (VGCCs). Whether and how VGCCs participate in the electrotaxis of glioblastoma may shed new insights for inhibiting glioblastoma infiltration.

VGCCs are known to play important roles in glioma biology such as cell proliferation, apoptosis, and sensitization to ionizing radiation.^27,83,84^ VGCCs can be categorized as high voltage activated (HVA) or low voltage activated (LVA) types.^85–87^ Among the HVA VGCCs, four subtypes can be categorized by the electrophysiological property and genetic phylogeny, including L-type (long-lasting, ${\text{Ca}}_{v}$ 1.1–1.4), P/Q-type (purkinje/unknown, ${\text{Ca}}_{v}$ 2.1), N-type (neural, ${\text{Ca}}_{v}$ 2.2), and R-type (residual, ${\text{Ca}}_{v}$ 2.3) VGCCs. The LVA VGCCs are composed of T-type channels (transient, ${\text{Ca}}_{v}$ 3.1–3.3). Although the involvement of VGCCs in the electrotaxis of glioblastoma is not yet elucidated, VGCCs' role in electrotaxis of other cell types has been proposed. Trollinger *et al.*, found that a stretch-sensitive VGCC mediates the electrotaxis of human keratinocytes.^88^ Aonuma *et al.*, reported that T-type VGCC mediates the electrotaxis of green paramecia.^89^ L-type VGCCs also regulate the chondrogenesis during early limb development which is known to be a bioelectricity process.^90^

Another class of membrane proteins that are bioelectrically activated is voltage-gated potassium channels (VGKCs), which are represented by 12 families ($K_{v}$ 1–$K_{v}$ 12). VGKCs are involved in diverse physiological and pathological processes regulating the repolarization of neuromuscular action potential, calcium homeostasis, cellular proliferation, migration, and cancer proliferation.^91–97^ Voltage-gated potassium channel $K_{v}$ 1.2^98^ and non voltage-gated inwardly rectifying potassium channel $K_{\text{ir}}$ 4.2^99^ have been shown to be involved in the sensing of electric field and signaling of cell electrotaxis. The potassium ion transporters confer biophysical signals that are key for regulating stem cells and tumor cell behavior in the microenvironment.^100^ In prostate cancer cells, VGKC expressions are linked to the increased metastatic potential.^101^ Furthermore, inhibition of $K_{v}$ 1.3 VGKC has been shown to induce apoptosis of glioblastoma cells *in vitro.*^102^

The role of VGCCs in glioblastoma cell electrotaxis is investigated by using pharmacological inhibitors (Table S3). Many of these inhibitors are short peptides purified or recombinant engineered from venoms of poisonous species. When testing inhibition, cytochrome C was added to the culture media to prevent non-specific adsorption of peptide inhibitors to microfluidic chips.^103^ The detailed results of pharmacological VGCC inhibition on the electrotaxis of T98G and U-251MG cells are shown in Figs. 4(a), S9, S10, and Table S4. The working concentrations of pharmacological inhibitors do not impact cell viability or random cell migration compared to control groups in 10% FBS (Fig. S7 and Table S4).

In both glioblastoma cell lines, inhibition of L-type HVA VGCC with gadolinium^104^ or nicardipine^105^ exhibits no effect on electrotactic directedness (P > 0.99) nor on speed (P > 0.15) (Fig. S9). Inhibition of N-type HVA VGCCs with *ω*-Conotoxin GVIA^106,107^ also has no effect on the electrotaxis of either cell type (P > 0.92).

#### 1. T98G electrotaxis is mediated by R-type HVA VGCC

The electrotaxis of T98G is repressed when treated with cadmium that is a broad spectrum HVA VGCC inhibitor at 50 *μ*M and 100 *μ*M [P < 0.0001, Fig. 4(a)].^108,109^ Upon further identification, the directedness in T98G electrotaxis is repressed by use of SNX482, an R-type VGCC inhibitor^110,111^ (P = 0.049). The direction tendency is decreased by the presence of SNX482 as presented by directional statistics (P=0.002, Mardia–Watson–Wheeler test) [Fig. 4(b)]. The electrotaxis of T98G cells repressed with SNX482 is shown in Video S5.

Calmodulin, a calcium binding protein, mediates many of the Ca^2+^ dependent-signaling by interacting with VGCCs and maintaining intracellular calcium homeostasis.^112,113^ However, the electrotaxis of T98G is not dependent on calmodulin by inhibition with calmidazolium [P > 0.99, Fig. 4(a)] and Ni^2+^ treatment has no inhibition on T98G cells (P > 0.99), which has partial inhibition on R-type VGCC.^111,114^ These results imply that an alternative mechanism might be at play.

#### 2. U-251MG electrotaxis is mediated by P/Q-type HVA VGCCs

U-251MG electrotaxis has exhibited its dependency on HVA VGCCs. Decreased directedness and speed are observed in U-251MG cells treated with 100 *μ*M cadmium (P = 0.0372, Table S4). Upon further identification, U-251MG electrotaxis directedness is repressed when treated with P/Q-type HVA VGCC inhibitor using agatoxin IVA and conotoxin MVIIC (P < 0.0001) [Fig. 4(a)]. However, the electrotactic speed is not affected by agatoxin IVA (P > 0.99) but decreased by conotoxin MVIIC (P < 0.0001) (Fig. S10). The electrotactic directedness of U-251MG is dependent on calmodulin (P < 0.0001). The electrotaxis of U-251MG is repressed by the treatment of agatoxin IVA, shown in Video S6. The direction tendency of U-251MG electrotaxis is decreased by the presence of *ω*-conotoxin MVIIC as presented by directional statistics (P < 1E-12, Mardia–Watson–Wheeler test) [Fig. 4(d)].

Furthermore, the electrotactic directedness of U-251MG cells is suppressedd with nickel (P < 0.0001) and amiloride (P < 0.01) as well as the electrotactic speed (P < 0.0001) [Figs. 4(a) and S10]. These results suggest a possible involvement of T-type VGCC^115^ in U-251MG electrotaxis. However, U-251MG electrotaxis is not affected when tested using another potent T-type VGCC inhibitor, TTA-A2.^116,117^

#### 3. T98G electrotaxis and U-251MG electrotaxis are also mediated by A-type VGKCs and acid-sensing sodium channels

In T98G electrotaxis, cadmium and SNX482 inhibit the R-type HVA VGCC and decrease the electrotaxis directedness. However, cadmium and SNX482 have also been reported to block rapid inactivating (A-type) transient outward VGKC ($K_{v}$ 4.3) so experimental results of SNX482 should be interpreted carefully.^118–120^ Using 5 *μ*M AmmTx3, a member of the *α*-KTX15 family of scorpion toxins, to block A-type VGKCs ($K_{v}$ 4.2 and $K_{v}$ 4.3),^121–123^ T98G electrotaxis directedness (FBS vs AmmTx3 of –0.69 vs –0.39, P = 0.0025) and speed (FBS vs AmmTx3 of 9.99 vs 4.42, P<0.0001) are repressed but not completely abolished (Table S4). Furthermore, the inhibition of T98G directeness caused by SNX482 is stronger than those by AmmTx3 (SNX482 vs AmmTx3 of –0.27 vs –0.39, P = 0.039, two-tailed t test). Another broad spectrum transient VGKC inhibitor, 4-aminopyridine (4-AP), is used to confirm the results from AmmTx3.^124–126^ Under 1 mM 4-AP, the directedness and speed in T98G electrotaxis are both repressed (P < 0.0001, Fig. 4). Increasing the concentration of 4-AP to 4 mM, even though electrotactic directedness has not changed (P > 0.99) compared to the control case, the migration speed is decreased (P < 0.0001). This is likely an artifact caused by part of T98G cells detaching from the surface rather than from the effect of actual electrotaxis (Fig. S11). These results suggest that T98G electrotaxis may be mediated by A-type VGKCs ($K_{v}$ 4.3), but the involvement of R-type VGCCs cannot be completely ruled out. A-type VGKC is also known to regulate calcium flux and resting membrane potential in astrocytes^127,128^ and therefore may interact with calcium channels during glioblastoma electrotaxis. The directional tendency of T98G electrotaxis is decreased by inhibiting A-type VGKC as presented by directional statistics (P < 1E-12, Mardia–Watson–Wheeler test) [Fig. 4(c)].

Similar inhibition of A-type VGKC also represses U-251MG electrotaxis. When U-251MG cells are treated with AmmTx3 and 4-AP, the electrotaxis directedness is inhibited by both compounds (P < 0.0001) and the speed is repressed in only 4-AP (P < 0.0001) but not AmmTx3 (P = 0.09) (Fig. S10). The directional tendency of U-251MG electrotaxis is also decreased by inhibiting A-type VGKC as presented by directional statistics (P < 1E-12, Mardia–Watson–Wheeler test) [Fig. 4(e)]. The electrotaxis of T98G and U-251MG is suppressed when A-type VGKC is inhibited through 1 mM 4-AP (more details shown in Videos S7 and S8). Further molecular studies to explore the roles of VGCCs and VGKCs in glioblastoma electrotaxis are important for future studies.

Furthermore, nickel and amiloride repress the directedness of U-251MG electrotaxis but not through T-type VGCC. At a high concentration of nickel and amiloride, the compounds may inhibit the acid-sensing ion channel (ASIC) and epithelial sodium channel (ENaC), which are members of a superfamily of voltage-insensitive mechanosensitive sodium channels.^129,130^ Furthermore, ASIC sodium channels are specifically expressed in the high-grade glioma cells but not in normal brain tissues or low grade glial cells.^131,132^ Sodium ion flux is known to mediate electrotaxis in keratinocytes through ENaC sodium channels^133^ and prostate cancer cells through voltage-gated sodium channels.^134–136^

To confirm the involvement of ASIC sodium channels in U-251MG electrotaxis, U-251MG cells are treated with 5 *μ*M benzamil hydrochloride (Alomone labs, USA).^137–139^ The directedness of U-251MG electrotaxis is significantly repressed by benzamil treatment [FBS vs benzamil = 0.58 vs 0.29, P < 0.0001, Fig. 4(a)] but not the speed (P = 0.42) (more details in Fig. S10). Benzamil is also tested on T98G electrotaxis and found to inhibit its directedness (P < 0.01) and speed (P < 0.0001).

#### 4. Various ion channels participate in the electrotaxis of glioblastoma cells of different origins

The pharmacological studies on the ion channels in T98G and U-251MG electrotaxis suggest that multiple ion channels, which may be voltage-sensitive or not, can mediate the sensing of endogenous electric field and initiate the migratory response.^13^ The proposed mechanism is highlighted in Fig. 5.

A-type VGKC, R-type VGCC, and ASIC sodium channels mediate the electrotaxis of T98G cells while P/Q-type VGCCs, A-type VGKC, and ASIC sodium channels mediate the electrotaxis of U-251MG cells. These results suggest that ion channel expression profiles are cell line specific and correlating ion channel expressions with electrotactic phenotypes of cancer cells may be beneficial to provide new insights of metastasis-aimed therapeutics by inhibiting electrotaxis.^140,141^ If glioblastoma infiltration can be inhibited by targeted therapeutics, the quality-of-life and prognosis of glioblastoma patients could be improved. The downstream molecular signaling of VGCCs, VGKCs, and ASIC sodium channels in glioblastoma electrotaxis is an interesting future direction to investigate. Recently, glutamatergic receptors have also been shown to mediate neuron-glioma interaction and glioma progression through calcium signaling.^142–144^ Therefore, a systematic screening of potassium channel, sodium channel, and glutamate receptor ion channels' ability to mediate glioblastoma electrotaxis is necessary to map the signaling network that may contribute to glioblastoma metastasis.

Our results together with previous reports^15,16,19,41^ suggest that electrotaxis may be an ensemble phenotype contributed by many signaling pathways, including chemical signaling, mechanosensing, and electrical activation (Fig. S1). The electrotaxis behavior may be highly dependent on the gene expression, membrane receptor composition,^19^ and assembly of signaling complexes in lipid rafts.^145^ Therefore, phenotypic characterization of cell electrotaxis and identification of membrane receptor compositions may help identify important molecular targets in a personalized medicine approach given that induced migration by physiological electric field may contribute to intracranial metastasis. The multiplex hybrid microdevices and machine learning-assisted quantitative analysis developed in this work can be very useful for systematic phenotype profiling and identification of molecular mechanisms underlying cell electrotaxis.

## III. CONCLUSION

As proof of principle, the hybrid PMMA/PDMS microfluidic chip demonstrates robustness and versatility for multiplex electrotaxis studies. Cell migration in multiple dcEFs under multiplex conditions can be studied in a single experiment in combination with automated microscopy. The submerged operation balancing the inlet/outlet hydrostatic pressures guarantees a stable microenvironment that avoids microbubbles, ensures uniform cell seeding, and minimizes required number of cells. Uniformly distributed single cells can be reliably seeded in a microfluidic chip that further increases the robustness and reproducibility of multiplex experiments. Use of machine learning-enabled single cell migration analysis automates the cell migration data analysis workflow for reliable quantitative data at high throughput, opening new opportunities for quantitatively studying cell responses in well-controlled microenvironments.

Geltrex^TM^ coating has been identified to support a reproducible electrotaxis model of T98G and U-251MG glioblastoma cells. The heterogeneity responses of T98G and U-251MG electrotaxis and the importance of calcium signaling are identified. By further inhibitorial study, the electrotaxis of T98G may depend on R-type HVA VGCCs, A-type VGKCs, and ASIC sodium channels. The electrotaxis of U-251MG depends on P/Q-type HVA VGCCs, A-type VGKCS, and ASIC sodium channels. Multiple ion channels, which may be voltage-sensitive or not, can mediate the sensing of electric field and electrotaxis in different glioblastoma models, suggesting that glioblastoma infiltration can be amplified by endogenous electric field in a tumor sample-dependent manner. The roles of ion channels in glioma metastasis and survival with regard to physiological electric field require further systematic studies and *in vivo* validation.

## IV. METHODS

### A. Hybrid multiple electric field device (HMEFC) design, simulation, and fabrication

Using the hybrid PMMA/PDMS approach, the prototyping disadvantages in both materials can be mitigated while the advantages can be combined. In PMMA, complex 3D structures for fluidic routing or reservoir and world-to-chip interface can be quickly prototyped by CO_2_ laser cutting and thermal bonding. However, spatial resolution using this approach is not sufficiently high to create reliable microfluidic environments. In contrast, precise quasi-two dimensional microstructures can be fabricated using the soft lithography technique in PDMS microfluidic chips. But standard soft lithography for PDMS fluidics is limited in the 3D design and world-to-chip interface. By using a dual-energy double-sided tape, PMMA and PDMS substrates can be easily and reversibly bonded,^17,146^ enabling broad experimental flexibility.

In HMEFC, two PMMA components for a world-to-chip interface and electrical application were adhered to a PDMS chip that contained a double-layer microchannel network where cells were cultured in and observed [Fig. 1(b)]. By double-layer microchannel design [Fig. 1(c)], experiments with two different cell types or chemical treatments with four electric field strength (EFS) conditions could be performed in a single chip.

To create multiple dcEFs, the HMEFC was designed by a R-2R resistor ladder configuration^17,31,32^ to create theoretical 5.25:2.5:1:0-ratio multiple EFs in Secs. I–IV and V–VIII [Fig. 1(c)]. The cells exposed to the highest dcEF were closest to the outlets to avoid paracrine signaling from electrically stimulated cells to un-stimulated cells.

By using the double-layer design, the hydraulic resistances in the 10 *μ*m-high, 0.84 mm-wide interconnecting channels were much higher than the two 100 *μ*m-high, 2 mm-wide main channels where cells resided, limiting the advectional chemical transport and avoiding “cross-contamination” events that further increased the high experimental throughput (see detailed discussion on chip design in the supplementary material).

Coupled numerical simulation of electric field, creeping flow, and chemical transport was carried out by finite element methods (COMSOL Multiphysics 5.3, COMSOL, USA). To correctly simulate the system, in-house measured material properties of the minimum essential media *α* (MEM*α*) supplemented with 10% FBS medium were measured and input in COMSOL. The liquid material properties of the 3D model were set as water with a density of 1002.9 Kg m^3^, an electrical conductivity of 1.536 S m^−1^, a dynamic viscosity of 0.946 mPa s, and a relative permittivity of 80. The numerically simulated electric field ratio was 4.99: 2.45:1:0 in Secs. I–IV and V–VIII with limited chemical transport across the interconnecting channels as designed (Fig. S15).

To fabricate a HMEFC, first, the PDMS chip was fabricated by a soft lithography technique.^147^ The double-layer microfluidic design was fabricated into a mold using negative photoresists (10 *μ*m and 100 *μ*m) on a silicon wafer using direct-write lithographic writer and mask aligner (DL-1000, Nano Systems Solution, Japan and MA/BA6, SUSS MicroTec, Germany). After passivation of the mold with perfluorosilane, mixed PDMS monomer (10:1 monomer:curing agent ratio, Sylgard 184, Corning, USA) was poured and cured on the master mold in a custom-made casting block, which ensures the 4 mm thickness in finished PDMS devices. After degassing, a piece of 15 mm-thick PMMA block was placed on top of the casting block to ensure the surface flatness of PDMS. The PDMS was cured in an oven and cut to yield individual devices. 1 mm-wide inlets and outlets were punched on cured PDMS devices and the PDMS devices were bonded to cover glasses (0.17 mm-thick) using O_2_ plasma, completing the PDMS chip.

Second, the PMMA components were fabricated by cutting the four-layer design on a 2-mm thick cast PMMA substrate (Kanase, Japan) using a CO_2_ laser cutter (VLS3.50, Universal Laser Systems, USA). The layers were aligned and thermally bonded on a programmable automated hot press with temperature and pressure control (G-12RS2000, Orihara Industrial Co., ltd, Japan). Third, dual energy double-sided tape (No.5302A, Nitto, Japan) was patterned using the CO_2_ laser cutter and used to join the PMMA components and the PDMS chip.^17,146^ A PMMA top reservoir for submerged cell seeding was also fabricated by four layers of 2 mm-thick PMMA pieces using laser cutting and thermal bonding. The PMMA top reservoir was affixed on top of the two PMMA components through PDMS padding frames with dual energy double sided tapes, completing the assembly of HMEFC for cell seeding.

The detailed description for the HMEFC design, simulation, and fabrication can be found in the supplementary material.

### B. Cell culture and maintenance

Glioblastoma cell lines T98G (CRL-1690, ATCC, USA) and U-251MG (IFO50288, JCRB, Japan) were obtained from the respective tissue banks and thawed according to the received instructions. Ethics approval is not required. T98G and U-251MG were cultured in minimum essential media *α* (MEM*α*) supplemented with 10% FBS and 2.2 g L^−1^ under 37 °C, 5% CO_2_ moist atmosphere (MCO-18AIC, Sanyo, Japan). The cells were subcultured every other day or whenever cells reached 80% confluency. Mycoplasma contamination was checked every three months using a mycoplasma species-specific PCR kit (e-Myco plus, iNtRON, Korea) on a thermocycler (C1000, Bio-Rad, USA).

Frozen stocks of the cells were prepared by resuspending 1 $\times 10^{6}$ log-phase cells in 1 ml CellBanker solution (Takara Bio, Japan) and cooled down in a freezing container (Mr. Frosty, Nunc, USA) in −80 °C overnight. The frozen cells were then transferred into the gaseous phase of liquid nitrogen for long term storage (Locator 6, Thermo Scientific, USA).

### C. Cell viability testing and senescence staining

The viability of cells under various media conditions and different chemical concentrations was tested using alamarBlue reduction assay (BUF012B, Bio-Rad, USA). The T98G and U-251MG cells were seeded in 96-well microplate wells at the number of 5 $\times 10^{3}$ log-phase cells per well. Each experimental condition was performed in triplicate where cells were incubated with different chemicals at working concentrations (Table S3) for 12 h before alamarBlue was further added and incubated for 4 h at 37 °C. Optical absorbances at 570 and 600 nm were measured on a microplate reader (Multiskan GO, ThermoFisher Scientific, USA). The viability was calculated based on the manufacturer's instructions and compared to that of the control set of cells in the media containing 10% FBS.

T98G cells have large and multinuclei subpopulations which have been proposed to play a role of drug resistance.^57,58^ The senescence-associated *β*-galactosidase activity in T98G cells was investigated following manufacturer's instructions (9860, Cell Signaling Technology, USA). T98G cells conditioned with 50 *μ*M hydrogen peroxide for 3 h and subcultured for 2 days were used as a positive control.^148^

### D. Cell seeding and electrotaxis experiment

The cell experiment workflow included salt bridge preparation, priming of microchannels, coating substrate with ECM, seeding cells, assembly of world-to-chip interface, and electrotaxis experiment.

First, sterilized 1% molten agarose (Seakem LE agarose, Lonza, USA) dissolved in 1X phosphate buffered saline (PBS) was injected on the salt bridge junctions of the PMMA component B and allowed to gel [Fig. 1(b)]. The salt bridge served as a solid electroconductive separation in electrotaxis experiments between the cell culture media and electrode, avoiding formation of complex electrolysis products.

Second, 50 *μ*l 99.5% ethanol (Wako, Japan) loaded in 200 *μ*l pipet tips were used to wet the microchannels in the PDMS chip by capillary flow and gravity flow.^17,149^ The microchannels were then washed with ultrapure water. The inlet/outlet ports were submerged under liquid solutions in all steps afterward to ensure bubble-free microchannels.

Third, 150 *μ*l of appropriate ECM solutions such as Geltrex^TM^ was loaded in tips and inserted on one side of inlet/outlet ports. The ECM solutions were allowed to flow into chip passively and incubated for 1 h at 37 °C. After ECM coatings, the channels were washed once with PBS and MEM*α*. The top reservoir was then filled with MEM*α* until the inlets and outlets were under the same liquid level, balancing the inlet/outlet hydrostatic pressure.

Log-phase glioblastoma cells were washed with 1X PBS, trypsinized (TrypLE, Thermo Fisher Scientific, USA), counted on a benchtop flow cytometer (Muse counting and viability kit, Millipore, USA), centrifuged at 300×g for 5 min, and resuspended in MEM*α* media with 10% FBS at 10^6^ cells ml^−1^. An appropriate amount of cell suspension was injected into the microchannels using a 200 *μ*l micropipet. Due to the small volume in microchannels, only a minute amount of cell suspension was needed. The cells were allowed to adhere in the chip under a 37 °C, 5% CO_2_ moist atmosphere for 3–5 h.

After cell seeding and adhesion, the top reservoir was removed for optical clarity. A piece of 4 mm-thick PDMS slab punched with two outlet holes (21 G, Accu-punch MP, Syneo Corp, USA) was affixed to the top of component B through the first piece of patterned double-sided tape [Fig. S17(a)], creating an air-tight seal.

To start the electrotaxis experiment, fresh media were supplied in the reservoirs on component A of HMEFC. Two sets of tubings (06419–01, Cole Parmer, USA) with stainless tubes (21RW, New England Small Tubes, USA) on one ends and double Luer gel dispensing needles on the opposite ends (23G, Musashi, Japan) were used. The tubings were sterilized before priming with 1X PBS with 2.5 ml syringes (Terumo, Japan). The stainless tube ends were inserted into the 21G holes of the PDMS slab on the HMEFC. The syringes were mounted on a syringe pump (YSP-202, YMC, Japan) and set in withdrawal mode to perfuse the cells on HMEFC. The HMEFC was completed and ready for the electrotaxis experiment [Fig. 1(b)].

Two HMEFCs were prepared in one experiment that allowed 16 conditions to be screened simultaneously. HMEFCs were affixed in a microscope on-stage incubator (WKSM, Tokai-hit, Japan). A feedback thin-film K-type thermocouple was attached to the glass bottom of a HMEFC (60 *μ*m-thick, Anbesmt, Japan) and regulate the incubator to maintain the environmental temperature at 37 °C.

A U-shaped PMMA salt bridge filled with 1% molten agarose in PBS was used to connect the two HMEFCs electrically by inserting into the ajoining PBS reservoirs on each HMEFC's component B. 300 V m^−1^ dcEFs were established in Secs. I and V [Fig. 1(c)] through two home-made silver/silver chloride (Ag/AgCl) wire electrodes^31,49^ inserted in the PBS reservoirs on component B by a source measure unit (SMU 2410, Keithley, USA). Time lapse phase contrast images of each condition were taken on an automated phase contrast microscope (Ti-E, Nikon, Japan). The setup is shown in Fig. S12.

For electrotaxis of glioblastoma on different ECM coatings, different ECM coatings were coated in the microchannels and electrotaxis experiments were performed in MEM*α* with 10% FBS for 6 h.

To test if the electrotaxis of gliblastoma cells require extracellular Ca^2+^, calcium-free Dulbecco's minimum essential medium (CaFree DMEM, 21068, Gibco, USA) or cation chelators such as ethylenediaminetetraacetic acid (EDTA) (15575, Thermo Fisher Scientific, USA) and ethylene glycol-bis(2-aminoethylether)-N,N,N′,N′-tetraacetic acid (EGTA) (08907–84, Nacalai, Japan) were used (Table S3). Before dcEF stimulation, cells were incubated in CaFree DMEM or MEM*α* with 10% FBS mixed with calcium chelators at indicated molar concentrations for 2 h at 20 *μ*l h^−1^. Afterward a direct electric current was applied through Ag/AgCl wire electrodes in D-PBS buffer on the PMMA component B by the source measure unit and cell behaviors were observed for six hours.

To investigate if voltage-gated ion channels mediate the electrotaxis of glioblastoma cells, the inhibitors were added to fresh MEM*α* media with 10% FBS at appropriate working concentrations (Table S3). The reagents were supplied in the reservoirs on the PMMA component A of HMEFCs after cells were seeded. The inhibitor-containing media were infused into the channels at 20 *μ*l min^−1^ for 10 min before changing to 20 *μ*l h^−1^ and incubated for 30 min. A direct electric current was applied through Ag/AgCl wire electrodes in PBS buffer on the PMMA component B by the source measure unit and cell behaviors were observed for six hours.

### E. Microscopy imaging and data processing

A Nikon Ti-E automated microscope with a Perfect Focus System and motorized XYZ stage was used to perform all microscopy experiments. A 10× phase contrast objective and intermediate magnification of 1.5× were used for taking 10-min interval phase contrast images with a scientific CMOS (sCMOS) camera with 2 × 2 binning (Orca Flash 4.0, Hamamatsu, Japan) in NIS Element AR software (Nikon, Japan). The spatial resolution at this setting was ≈ 0.87 *μ*m pixel^–1^. All experiments were performed in triplicate.

After each experiment, images were exported from NIS element software as tiff files and automatically organized by XY positions into folders using an in-house developed Python script. The cells in the raw images were segmented, tracked, and automatically analyzed using the *Usiigaci* software.^30^ Only cells tracked throughout all the frames in one viewfield were analyzed. At least 100 cells in every conditions were analyzed for cell-centric features such as the directedness, speed, and orientation changes before and after electric field or chemical stimulation (Table S5).

Briefly, the definitions of key cell-centric features used to quantify cell electrotaxis (Fig. S13) were listed below:

• Area The cell area was extracted as the average area (*μ*m^2^) which was integrated from individual cell masks, $\sum_{i=1}^{N}\frac{A_{i}}{N}$, where *A_i_* was the area of each cell, and *N* was the total number of analyzed cells.
• Directedness
  The directedness of cell electrotaxis was defined as the average cosine of Euclidean vector and EF vector, $\sum_{i=1}^{N}\frac{\;\text{cos}\;\Phi_{i}}{N}$, where $\Phi_{i}$ was the angle between the Euclidean vector of each cell migration and the vector of applied EF (from anode to cathode), and *N* was the total number of analyzed cells. A group of anodal moving cells held a directedness of −1; and a group of cathodal moving cells held a directedness of +1. For a group of randomly migrating cells, the directedness was zero.
• Speed
  The speed of cell electrotaxis was defined as the average of cell migration rate to travel the Euclidean distance, $\sum_{i=1}^{N}\frac{{\left(\frac{d_{\mathrm{net}}}{t_{\mathrm{elapsed}}}\right)}_{i}}{N}$, where the *d_net_* was the Euclidean distance traveled by each cell, and *t_elapsed_* was the time elapsed, and *N* was the total number of analyzed cells.
• Orientation
  The orientation was defined as the average cosine of two times angle between the EF vector and the cell long axis, $\sum_{i=1}^{N}\frac{\;\text{cos}\;2\theta_{i}}{N}$, where *θ_i_* was the angle between the applied EF vector and the long axis of a given cell; *N* is the total number of cells analyzed. A group of cells aligned perpendicular to the EF held an orientation of −1; and a group of cells aligned in parallel to the applied EF held an orientation of +1. For a group of randomly shaped cells, the average orientation was zero.

The cell-centric features were computed automatically and saved as Excel spreadsheets (Office365, Microsoft, USA) as part of the *Usiigaci* software analysis pipeline. The data were further statistically inferenced by inputting the data in statistical software (Prism 7, GraphPad LLC, USA). All data were presented as the mean ± 95% confidence interval, which was 1.96 of standard error of mean, from triplicate experiments. Two-tailed Student's t tests or one-way analyses of variance (ANOVA) with Tukey's multiple-comparison post-hoc tests were performed for statistical testing between two groups or multiple groups. The results from one-way ANOVA were reported until noted otherwise. The confidence level to reject a null hypothesis between two datasets was set at 95%. A p-value (P, the probability for a true null hypothesis) less than 0.05 represented a statistical significance at 95% confidence.

The directional tendencies of glioblastoma electrotaxis were also analyzed in directional statistics (Oriana 4, USA). The X-Y displacements of glioblastoma cell electrotaxis after 6 h were calculated into angles by trigonometry and plotted in angle histogram plots (rose plots). A non-parametric Mardia–Watson–Wheeler test was performed to test if the migration tendencies of two groups were identical.^150^ The P value less than 0.05 represented confidence to reject null hypothesis at 95% confidence level.

## SUPPLEMENTARY MATERIAL

See the supplementary material for the supplementary figures, supplementary tables, supplementary videos, and detailed description of HMEFC chip design, simulation, and fabrication.

## Data Availability

The data that support the findings of this study are available from the corresponding author upon reasonable request.
